# Supplementary material for: Heterologous Expression, Purification, and Immunomodulatory Effects of Recombinant Lipoprotein GUDIV-103 Isolated from Ureaplasma diversum
Source: Microorganisms. 2022 May 16;10(5):1032. doi: 10.3390/microorganisms10051032 (PMC9147684; doi:10.3390/microorganisms10051032)
Supplement: Supplementary file 1 [file microorganisms-10-01032-s001.zip › microorganisms-1705492-supplementary.pdf]

Supplementary Materials

Table S1: *Ureaplasma diversum* isolates from different farms and Brazilian states

Isolate	Location		Isolate	Location	
	Farm	State *		Farm	State *
**BA78**	10	BA	**805**	3	MS
**34**	10	BA	**9653**	3	MS
**35**	10	BA	**A203**	1	SP
**37**	10	BA	**GOTA**	2	SP
**47**	11	BA	**S6**	9	SP
**51**	11	BA	**S8**	9	SP
**52**	11	BA	**5T**	8	SP
**54**	11	BA	**7T**	8	SP
**55**	11	BA	**10T**	8	SP
**56**	11	BA	**13T**	8	SP
**78**	10	BA	**16T**	8	SP
**83**	10	BA	**72**	4	SP
**84,2**	10	BA	**73**	4	SP
**89**	10	BA	**84**	4	SP
**111**	13	BA	**93**	2	SP
**133**	13	BA	**94**	2	SP
**148**	13	BA	**95**	2	SP
**174**	13	BA	**98**	2	SP
**198**	13	BA	**100**	2	SP
**234**	12	BA	**102**	2	SP
**239**	12	BA	**59**	1	SP
**249**	12	BA	**A523****	–	–
**Ud18**	**6**	**MG**	**ATCC 49782*****	–	–

* BA: Bahia, MG: Minas Gerais, MS: Mato Grosso do Sul, SP: São Paulo. **No defined location. *** Reference strain isolated from a cow with granular vulvovaginitis in Ontario, Canada [39].

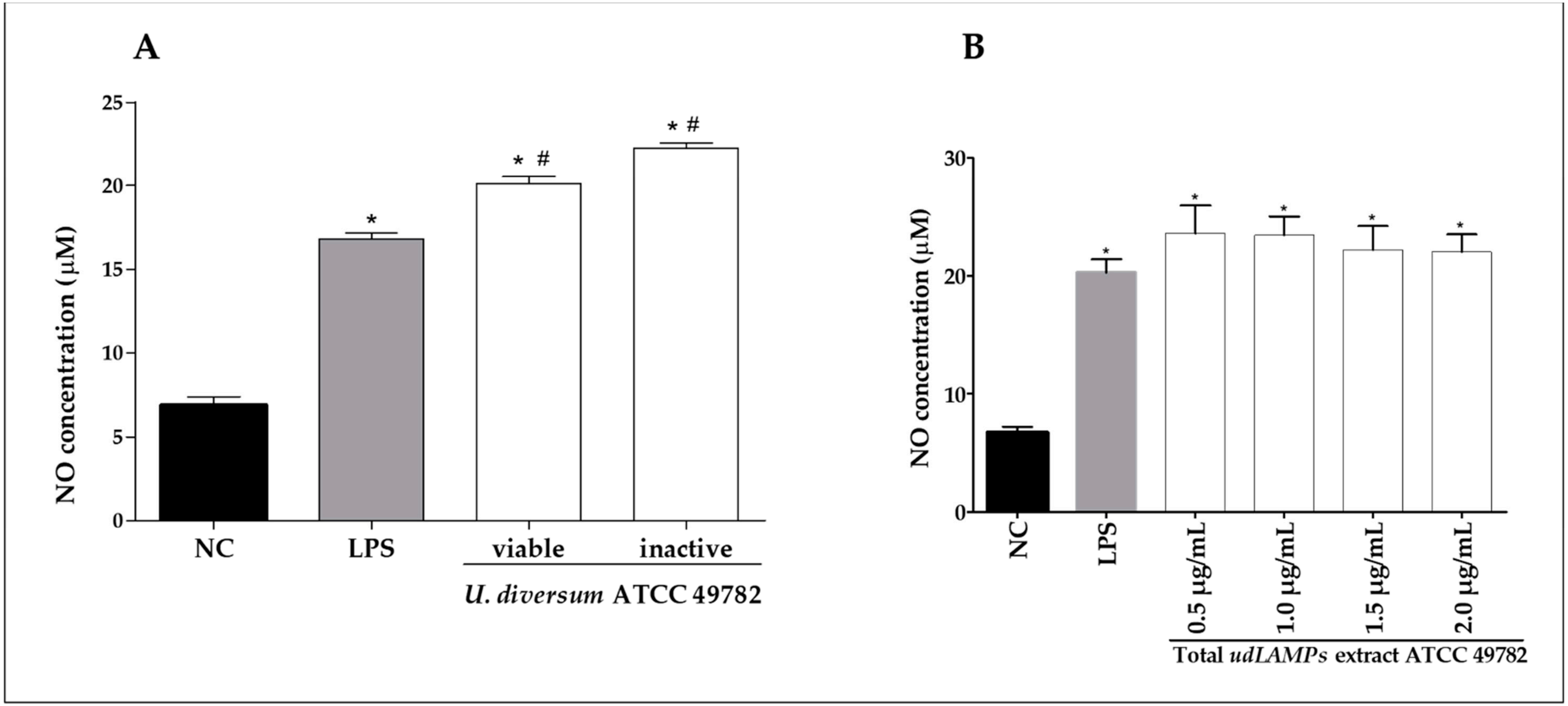

Figure S1. NO concentration induced in bovine peripheral blood mononuclear cell (PBMC) culture supernatant after incubation with viable and inactive *U. diversum* ATCC 49782 (A) and after incubation with 0.5, 1.0, 1,5 and 2.0 μg/mL of total lipoproteins extracted (B). Treatments were compared using the Kruskal–Wallis non-parametric test followed by Dunn's post-hoc test. Different symbols indicate statistically different groups. * $p < 0.05$ vs negative group. # $p < 0.05$ vs. LPS group. Data are expressed as the mean ± standard deviation (n = 9).
